# Transactions of Illinois State Medical Society, 1859

**Published:** 1860-01

**Authors:** 


TRANSACTIONS OF THE ILLINOIS STATE MEDICAL SOCIETY, held at Decatur, June 7th and 8th, 1859. Pages 146.

If the merits of a volume were to be judged of by its size, we should not be disposed to pronounce the Transactions as a very valuable contribution to our medical literature. But as we have progressed in the examination of its contents, our anxiety has given place to a feeling of satisfaction, that as meagre in size as it is, still the volume lacks none of the interest and merit of its predecessors.

After the usual proceedings, we find occupying the first few pages the Valedictory Address of the retiring President, in which was presented, in Dr. Johnson's peculiarly felicitous style, the subject of human dissection, and the interest which the people of any community should feel in the pursuit of anatomical studies. In answer to the various objections that are urged against the only successful mode of studying practical anatomy, the Doctor remarks, that "it was not mere curiosity that actuated the student in the daily pursuit of his favorite science, nor was it so much to meet the claims of abstract science; but that there was a necessity of a personal practical acquaintance with the structures of the human body, as the qualification for the successful discharge of the claims society is constantly demanding of the physician." And "as there are those who are bound to society by no ties but those of our common humanity—or are in our prisons and alms-houses—whose bodies are unclaimed, and whose graves will remain undistinguished—if by legislative enactments, authority should be granted to those having charge of such institutions, to deliver after death, the bodies of those who might remain uncared for, to organized medical colleges, and such regular physicians and surgeons as might apply for them, and as in the judgment of the authorities were persons of suitable discretion, the demands of society and the interests of humanity would be fully met, without doing violence to the feelings of surviving friends, or the natural respect which all men feel for the dead." Now, if this is the practical plan, it behooves every member of the profession and of society at large to early secure in some way the legalization of anatomical studies. And, then, no longer would we see the strange inconsistency of our laws, with reference to professional skill and qualification. Nor would there be laws by which every physician and surgeon is made liable to suits and heavy damages for mal-practice if he do not exercise skillfully that knowledge of practical anatomy, for the acquiring of which, he is now subjected to heavy penalties, and public sentiment deems him guilty of a high crime and his name is associated with felons and murderers. And, if instead of making the taking of bodies from the potters field a penitentiary crime, the next legislature would pass a law similar to one enacted and in operation in New York and Massachusetts, the public at large would rest in full security, cherishing the memory of their dead, and undisturbed by any fears as to the sanctity of their last resting place."

In conclusion, we have only to express a wish that every member of the next legislature of our State should be supplied with a copy of this excellent address, for it will exert a powerful influence in educating public sentiment, and in obviating the trivial and superstitious objections that are urged against the study of practical anatomy.

The next fourteen pages we find occupied by a report on the Sore-Mouth of Nursing Women, by J. H. Hollister, M. D., Chicago.

With a brief abstract of the literature of this affection, the writer passes to the consideration: 1st. That it is a disease of very general occurrence in some localities, while it is not known in others. 2d. That females alone suffer from it; and then only during the periods of gestation and lactation, and sometimes there is a recurrence after lactation has ceased. 3d. That the intensity of its development is very uniformly dependent upon an impoverished condition of the blood; and also a diminution of a red corpuscles—and that it assumes an epidemic form from a concurrence of influences which depress the vital powers. That its three forms are but the different manifestations of the same disease, and that the erythematous variety is migratory in its character; and any of the mucous membranes are liable to its metastasis, while the common complications are to be met with in the lungs, stomach, and bowels. In the treatment, the general indication is to be attained by a generous support of the vital powers by all the means at our command, and in addition to this, "the suppression of the lacteal secretion may be imperative."

The report of the Committee on Obstetrics and Diseases of Women and Children occupies some fifty pages, and is made up of several interesting original cases, and one essay on the use of "Chloroform in Parturition." The design of the Committee seems to have been "to make the report original, corresponding more nearly with the design of the appointment, while it is really adding something to medical literature." The arrangement of the subjects is such, that each case presents something of the distinctness of a separate report upon the cases presented; among which may be cited a case of Pelvic Abscess, by Dr. Byford, the chairman of the Committee; together with a singular case of extensive abscess of the ovarian region, by Dr. Noble. Cauliflower Excresence of the Os Uteri, with two cases. Placenta Previa, with case. A case of extensive wound of the abdomen during pregnancy, reported by Dr. S. H. Luce. We next find an interesting contribution from the fertile pen of Dr. A. Hard, of Aurora, of six cases of Monstrosities and Mal-formations, and one of Puerperal Convulsions, that presents the peculiarity of treatment by Ti. V. Viride, with free bleeding, by which means the pulse became slower and the labor progressed favorably. Then follows a communication of interesting cases of Puerperal Convulsions, complicating labor, by our fellow-townsman, Dr. V. L. Hurlbut. Ovarian Tumor, complicating labor, by the Chairman. Also a case in which there was a rupture of the uterus during the progress of labor. A case of the poisoning of the mother through the absorption of a decomposing fœtus, as furnished by Dr. J. H. Woodworth, of Chicago; and in contrast to which, is another case by the Chairman, in which the mother carried the fœtus 51 days after its death without any apparent inconvenience, and then had a good delivery; and the Doctor remarks that the fœtus had gone farther in decomposition than in the case of Dr. Woodworth's, but it was of the adipocerous nature.

In conclusion of the report we find a very interesting and well written essay on the use of Chloroform in Parturition, by Dr. Young. There is certainly no subject in the whole range of obstetrical practice of equal interest or importance. Under what circumstances should anæsthetic agents be administered to patients in the throes of labor? Should they be given in natural, or only in protracted instrumental cases? What are the dangers attending them, and how may they be most successfully guarded against? These, and many other questions, are continually presented to the mind of the practitioner, and the great mass of the profession are still in doubt and anxiety in regard to their solution. After discussing the safety and efficiency of chloroform, when judiciously administered; the various advantages that follows; the more perfect control of the uterus after delivery, and the consequent diminished liability to hemorrhage; the essayist concludes, that since the cases in which the anæsthetic agents have been administered are numbered by the thousand, and that few fatal cases, if any, have occurred, that it is sufficient to establish the safety as well as the power of anæsthesia, and should enable us to confer upon woman the greatest benefit she has ever received from medical science. In surgery, however, several unfortunate cases have occurred, that have also served as a caution in the use of anæsthetics in obstetrics. But we find an ingenious explanation of the different results furnished in the essay—"that as in surgery the agent is used as a preparation for the operation, pain is not at the time present, and has not exerted its influence upon the nervous system; but in parturition the pain is real and present, and not simply anticipated; therefore, the sensory portions of the nervous centre will resist its influence with more tenacity and force than if no pain was present."

The remainder of the volume is occupied by two Prize Essays. The first is on the influence of climate on Pulmonary Tuberculosis, and contains many facts of interest. It was written by Dr. G. W. Phillips, of Dixon, Lee Co., Ill. The second is on the use of Opium in the Treatment of Inflammatory Diseases, by Dr. A. S. Hudson, of Sterling, Ill. We shall defer a more full notice of these essays until a future number of the *Examiner*.

